# Public Private Partnership in Meghalaya: delivering healthcare in difficult to access tribal areas

**DOI:** 10.1186/1753-6561-6-S5-P3

**Published:** 2012-09-28

**Authors:** Dilip Singh Mairembam, Suchitra Lisam, Rajani Ved, Jhimly Barua, Prankul Goel, Roli Srivastava, Tushar Mokashi

**Affiliations:** 1 National Health Systems Resource Centre, New Delhi, India

## Introduction

Meghalaya, a hilly tribal state in northeast India, has many inaccessible areas with little or no access to good quality health services. With an infant mortality rate (IMR) of 58 infant deaths per 1000 live births, an institutional delivery proportion of 29.7 % of all deliveries, a full immunization coverage rate of 32.8 %, and a total fertility rate of 3.8, Meghalaya has some of the poorest health indicators in the country. Faced with challenges of attracting and retaining skilled health workers in these remote settings, the state adopted a Public Private Partnership (PPP) approach in 2008. The approach involves handing over the management and operations of 29 poorly performing community health centres (CHC), primary health centres (PHC) and subcentres located in difficult areas to non-governmental organisations (NGO), through a memorandum of understanding. We conducted this study to assess the effectiveness of this approach with respect to increasing access to essential healthcare services.

## Methods

We included all the 32 health facilities managed by the six NGOs (2 CHCs, 19 PHCs, 10 SCs and 1 dispensary) in the study. The study was conducted from November 2011 to January 2012. We used a mix of both quantitative and qualitative tools. We conducted a desk review of records and reports to assess performance of facilities. We supplemented this with observations made during facility visits to assess infrastructure development and maintenance, and human resources development. Focus group discussions were organized to assess community perceptions with respect to the village health committees and Accredited Social Health Activist (ASHA) programme. A pre-tested questionnaire was administered to service providers and a sample of beneficiaries.

## Results

We found improvement of most of the facilities and the functioning of the health centres in terms of service delivery following the takeover by NGOs. Improvements took the form of infrastructural corrections, better maintenance of buildings, improved staff availability, and better supply of essential drugs and equipment.

Since these facilities were performing near zero at the time of outsourcing management, the first major improvement is the recorded increase in outpatient care in all facilities and in-patient care in the CHCs. In the 21 health centres (2 CHC/19 PHC) of the 32 institutions we studied, every month 19362 outpatients and 907 inpatients were recorded. Antenatal coverage improved in 18 of the 19 sectors. Institutional deliveries are being conducted at all the 19 PHCs and two CHCs with an average of three to four deliveries per facility per month. Immunization sessions are being held regularly and number of fully immunized children has steadily increased. The main gaps were in skills of the providers and therefore, the quality of care. This was mainly due to lack of access to training programmes for the service providers.

## Discussion

With better management under PPP, previously non-operational health facilities located in difficult-to-access and underserved areas can deliver health care services. What helps the NGO succeed where the earlier system fails is a more flexible approach in recruitment, good leadership, building a supportive positive practice environment ensuring the retention of human resources.

If contracting-out management of health facilities in difficult-to-access areas is done in isolation from other state support functions, then the outcomes are limited. The State needs to supplement the facility level efforts with smooth flow of funds, need-based training programs, regular supply of drugs and logistics, and monitoring and support mechanisms. The state would also need to devise and implement incentive package for retention of key staff in these difficult and inaccessible areas.

The PHCs are being managed at a cost of around INR 2,200,000 per annum, which is the same as that spent on a regular PHC. The management requires support to be able to assess and manage the health of populations they serve, in addition to provision of healthcare services to those who come to the facility.

## Competing interests

None declared

## Funding statement

The study was funded by the National Health Systems Resource Centre, New Delhi and the Regional Resource Centre, Guwahati.

